# Pairing taVNS and CIMT is feasible and may improve upper extremity function in infants

**DOI:** 10.3389/fped.2024.1365767

**Published:** 2024-02-13

**Authors:** Kelly McGloon, Elizabeth Humanitzki, Julia Brennan, Philip Summers, Alyssa Brennan, Mark S. George, Bashar W. Badran, Anne R. Cribb, Dorothea Jenkins, Patricia Coker-Bolt

**Affiliations:** ^1^ Department of Rehabilitation Science, College of Health Professions, Medical University of South Carolina, Charleston, SC, United States; ^2^ Department of Health Science and Research, College of Health Professions, Medical University of South Carolina, Charleston, SC, United States; ^3^ Department of Psychiatry and Behavioral Sciences, Medical University of South Carolina, Charleston, SC, United States; ^4^ Department of Pediatrics, Medical University of South Carolina, Charleston, SC, United States; ^5^ Ralph H. Johnson VA Medical Center, Charleston, SC, United States; ^6^ College of Medicine, Medical University of South Carolina, Charleston, SC, United States

**Keywords:** cerebral palsy, taVNS, auricular nerve stimulation, motor rehabilitation, neuromodulation, vagus nerve

## Abstract

In this study we combined non-invasive transcutaneous auricular vagal nerve stimulation (taVNS) with 40 h of constraint induced movement therapy (CIMT) in infants. All infants completed the full intervention with no adverse events. Therapists were able to maintain high treatment fidelity and reported high ratings for ease of use and child tolerance. Preliminary results show promising gains on motor outcomes: Mean QUEST increase 19.17 (minimal clinically important difference, MCID 4.89); Mean GMFM increase 13.33 (MCID 1%–3%). Infants also exceeded expectations on Goal Attainment Scores (+1). Early data is promising that taVNS paired with intensive motor CIMT is feasible, reliable, and safe in young infants with hemiplegia, and may help harness activity-dependent plasticity to enhance functional movement.

## 1 Introduction

Perinatal central nervous system injuries may manifest in the first 12 months of life as developmental delays and motor weaknesses that presage hemiplegic cerebral palsy (CP) (1–3). Early rehabilitation takes advantage of critical windows of neuroplasticity in infants to ameliorate outcomes from these injuries (1, 4). Best practice motor interventions consist of both high doses and high intensity repetitions of real-life tasks with goal-directed practice (5), as in Constraint-Induced Movement Therapy (CIMT), one of the most effective treatments for children with hemiplegia (6). While the minimally effective CIMT dosage is 40 h (7), optimal dosage for lasting, functional motor changes requires 60–120 h (8). However, high dosage CIMT entails a significant time, resource and energy commitment from the therapist and family, and is difficult to incorporate into a family's typical day.

There is growing evidence that vagus nerve stimulation (VNS) delivered simultaneously with active motor interventions enhances and accelerates functional outcomes, motor learning and neuroplasticity (9–14). Adult human (9, 14) and animal studies (15) of implanted VNS administered with repetitive upper extremity practice show evidence of cortical motor neuronal reorganization and enhanced functional outcomes. A phase III pivotal trial of VNS paired with motor rehabilitation in adult stroke showed improved function when compared to motor training alone with minimal side effects, leading to FDA approval (16). Although promising, surgical implantation makes this technology prohibitive for many conditions and populations. A non-invasive VNS approach, transcutaneous auricular vagus nerve (taVNS), stimulates the auricular branch of the vagus nerve and mimics the CNS effects of implanted VNS when assessed by functional MRI (17–19). taVNS has shown motor facilitatory effects when paired with motor rehabilitation in adult stroke (20) and infants with perinatal brain injury (12, 21–23). We hypothesized that the addition of taVNS to CIMT may be beneficial for optimizing CIMT by harnessing early developmental neuroplasticity, accelerating CIMT-induced motor learning, and reducing the CIMT dose requirement from 3 months to 1 month to achieve persistent functional gains.

In this case series we investigated the feasibility and safety of taVNS paired with CIMT in 10–14-month-old infants with hemiplegia. Additionally, we determined the fidelity and quality of CIMT delivered while the therapist manually triggers taVNS, and the preliminary treatment effect on motor outcomes. Ensuring high CIMT fidelity in this therapy-intensive treatment is a critical aspect of evaluating the feasibility and efficiency of translating taVNS paired with CIMT into clinical settings (24). CIMT is one of the few therapeutic interventions that has a well-defined fidelity measurement tool, the Fidelity of Rehabilitation Implementation Measure (FIRM) (25). Establishing the validity and reproducibility of taVNS triggered with CIMT is important in moving this neuromodulatory treatment forward.

## 2 Methods

This pilot study was designed as an open-label study delivering taVNS pulses with movement of the hemiplegic limb during a 4-week, 40 h CIMT intervention. We monitored stimulation time, fidelity measures via FIRM scoring of recorded video sessions, and the infant's and therapist's tolerance of the device. Additionally, motor outcomes were measured before and after treatment, at 1 and 3 months. All study procedures were approved by the Medical University of South Carolina Institutional Review Board (IRB). We obtained written informed consent from all parents. This trial was registered on Clinicaltrials.gov (NCT05101707). Written informed consent was obtained from the parent for the publication of any potentially identifiable images or data included in this article.

### 2.1 Participants

We enrolled infants aged 6–18 months based on the following inclusion and exclusion criteria: *Inclusion Criteria:* Infants, 6–18 months old with hemiplegia/motor asymmetry after perinatal injury, with some active movement of the affected arm, and level I-IV function by the Gross Motor Function Classification System (GMFCS) (26). Prior CIMT therapy was allowed if delivered >3 months from enrollment. *Exclusion Criteria:* GMFCS level V, severe quadriplegic involvement, flaccidity of affected arm, uncorrected blindness or deafness, cardiomyopathy, inability to actively engage in 2 h of therapy, uncontrolled seizures, or CIMT therapy within the prior 3 months.

### 2.2 CIMT implementation

An occupational therapist and a physical therapist with >6 years experience were trained in the ACQUIRE intensive therapy framework (27), following the essential elements of a signature form of CIMT. The treating therapists participate in a multi-site randomized control trial (I-ACQUIRE). Our CIMT protocol aligned with the published infant CIMT protocols (28), including Baby CIMT (29) and Baby CHAMP (8), with regard to (1) the use of a constraint on a child's stronger arm, (2) the use of repetitive task practice and shaping techniques during sessions, (3) dosage higher than usual for a customary therapy intervention and (4) a transition plan provided to the family at the end of the program for maintenance of gains (30).

Therapists engaged infants in various activities to facilitate active, goal-directed movements. For example, the therapist would encourage infants to reach outside their base of support to knock over blocks to work on controlled weight shifting, shoulder flexion, and elbow extension. Each family was encouraged to participate in home-based activities on a daily/weekly basis during the treatment period with adherence documented at the start of each session. Families were provided a transition plan to encourage carry-over of bimanual skills following the end of the CIMT intervention. We did not specifically document each family's ability to follow through with the transition plan at 3-months. Each child and family continued their regular therapy services during the CIMT intervention.

### 2.3 Transcutaneous auricular vagus nerve stimulation (taVNS)

**Figure 1:**
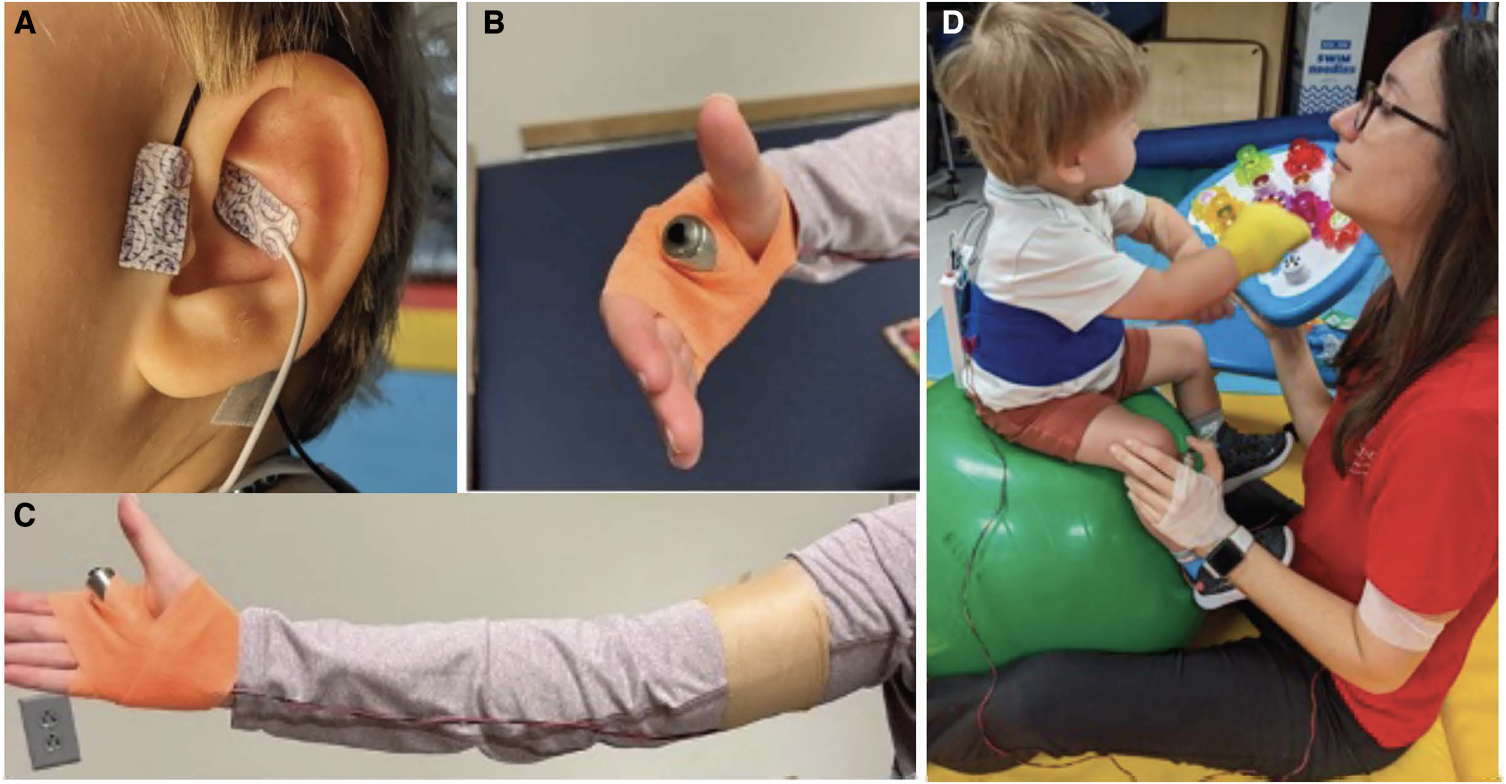
The custom taVNS set-up consisted of a taVNS unit (Soterix Medical, New York, NY) and adhesive hydrogel ear electrodes (Neotech, Valencia, CA) placed in front of the tragus and at the cymbae conchae (**A**) stimulation was triggered manually by the therapist to reinforce proper movements using a trigger button strapped to the hand (**B**,**C**). The taVNS unit was secured on the infant's back using a Velcro cummerbund but could be removed and placed on the mat for activities like rolling. The set-up is shown in treatment context (**D**).

A custom auricular stimulation system was designed using the Soterix electronic pulse generator unit (EPG, Soterix Medical, USA). The design involves a hand-held trigger that is connected to the external pulse generator. The hydrogel electrocardiogram leads (Micro Neoleads®, Neotech Products, USA) were used as ear electrodes and placed at the cymbae conchae and outer tragus to stimulate the auricular branch of the vagus nerve. The set-up of our taVNS system is shown in Figure 1.

Stimulation protocol: The electronic pulse generator delivered stimulation at a frequency of 25 Hz, pulse width of 500 ms, and current intensity at 0.1 mA below the level that the infants noticed the stimulation, termed the perceptual threshold (PT). We determined the PT before each session by increasing current in 0.1 mA increments until the infant detected the tingling feeling of the stimulation as evidenced by a facial grimace, shoulder shrug, reaching towards their ear, and/or vocalization. We paired taVNS at 0.1 mA less than the PT with CIMT after ensuring that the infant was not reacting to the stimulation at this level. This infant taVNS protocol was developed and used safely in over 700 stimulation sessions in infants enrolled in a feeding failure study (12, 13, 31). The EPG device displays the quality of the electrode connection (good, moderate, or poor), allowing adjustments to electrodes to be made in real-time to ensure stimulation is being delivered. Set-up took approximately 10–15 min.

Accurately pairing taVNS with the desired motor activity is crucial to the success of stimulation (32). The therapist manually triggered taVNS during active (1) arm/hand movement, (2) trunk activation during dynamic balance or gross motor tasks, or (3) attention to or tactile stimulation of the affected limb. Stimulation was paused when the infant stopped active engagement of the affected limb or at the end of a 2 min train, whichever period of time was shorter. An independent observer recorded active stimulation time.

Breaks were allowed within a session as necessary for infant tolerance. Therapists reported on the user experience for each session including notes for (1) infant's tolerance of the session, (2) ease of use of the taVNS system and (3) confidence in their ability to activate stimulation at appropriate times using a Likert scaling. All sessions were conducted at the Medical University of South Carolina.

### 2.4 Safety monitoring

Therapists examined the electrode site for redness or irritation at the beginning and end of each session. Within sessions, if a child demonstrated signs of stimulation awareness or discomfort, the PT was re-assessed or decreased by 0.1 mA until the child did not show signs of detecting stimulation and was able to return to participation in therapeutic activities. Parents were asked to report any changes in gastroesophageal reflux, irritability, sleep, and quality of vocalizations.

### 2.5 CIMT fidelity

Raters were trained in the CIMT Fidelity of Rehabilitation Implementation Measure (FIRM) which measures how consistently therapists used CIMT ACQUIRE principles and operant conditioning techniques. The FIRM is the updated version of the fidelity measure used in previously published studies (33). Two video recordings per week (40% of sessions) were selected at random and scored by two independent reviewers. Sessions were rated on a scale of 1–4. A score of 3–4 indicated the therapist's performance was consistent with established CIMT protocol expectations including selection of appropriate activities, cuing, etc. We averaged the 2 rater scores. There were no discrepancies in acceptable vs. unacceptable ratings between raters.

### 2.6 Motor outcomes

Motor outcome measures were collected at baseline, post-intervention, and at the 3-month follow-up visit. We used tests validated in children with cerebral palsy [Quality of Upper Extremity Skills Test (QUEST), the Gross Motor Function Measure-88 (GMFM-88)], the Developmental Assessment of Young Children (DAYC-2)]. The DAYC-2 is a general pediatric assessment used to screen for developmental delays and is not specific to children with CP.

Therapists used Goal Attainment Scaling (GAS) to collaboratively develop and set individualized goals that best fit the child's needs and parent goals/desires. Generally, 4 goals were established with 2–3 goals focusing on fine motor and upper extremity tasks and 1–2 goals addressing overall gross motor tasks.

GAS uses a 5-level incremental scale from −2 to +2 (−2, −1, 0, +1, +2) for each goal. The child's baseline ability is the base score of −2. The child's “expected progress” is a raw score of 0 (*t*-score = 50). A raw score < 0 (*t*-score < 50) indicates “less than the expected goal” was achieved. A raw score of >0 (*t*-score > 50) indicates “more than the expected goal” was achieved. Individual goal scores can be averaged to produce a cumulative score indicating overall intervention effectiveness.

To quantitatively establish an individual goal's scale, only one measured variable (time, number of repetitions) is set with an equal interval between raw scores. For example:

• Baseline (−2): While in a seated position with support at the upper abdominal muscles, child reaches for a target object **1 time (1x)** in one minute.
• Less than expected outcome (−1): **4x** in one minute.
• Expected outcome (0): child reaches for a target object **8x** in one minute.
• Greater than expected outcome (+1): **12x** in one minute.
• Much greater than expected outcome (+2): **16x** in one minute.

Our therapists have extensive expertise with the GAS and CIMT treatment as part of their primary clinical role and use their knowledge of the child's current abilities to design goals with appropriate, equal-level intervals to reach this “expected goal.”

## 3 Results

We enrolled three infants after obtaining parental consent. None of the infants had previously participated in CIMT. All 3 infants were White (not Hispanic/ Latino) male infants who had suffered antenatal or perinatal brain injuries. Further demographics are listed below.

• Infant 1 was a 10-month-old twin born at 23 weeks gestation without the benefit of antenatal steroids. He had significant clinical instability shortly after birth due to hypotension, coagulopathy, respiratory failure and pneumothoraces, and persistent pulmonary hypertension requiring inhaled nitric oxide. He experienced right grade 4 and left grade 2 intraventricular hemorrhages with post-hemorrhagic hydrocephalus and right porencephalic cyst formation. He had a ventriculoperitoneal shunt placed after repeated decompression via a reservoir failed to alleviate the increase in ventricular volumes. He had left-sided hemiplegia and deficits in sitting balance and transitional movements.
• Infant 2 was a 14-month-old born at 38 weeks gestation with an uneventful pregnancy and neonatal course. He showed early motor delays and MRI at 12 months revealed old left periventricular white matter infarctions, that extended into the corticospinal tracts at the cerebellar peduncles, presumably antenatal in origin but of undetermined etiology. He had right-sided hemiplegia and deficits in bimanual tasks and transitional movements.
• Infant 3 was a 12-month-old born emergently at 36 weeks gestation for fetal distress with a history of decreased fetal movement and biophysical profile of 2 out of a possible score of 8, indicating significant fetal compromise. He experienced moderate hypoxic ischemic encephalopathy and qualified for therapeutic hypothermia treatment for 72 h per standard protocol. MRI at 7 days of age showed bilateral frontal white matter infarcts with restricted diffusion, as well as a large lactate peak and very low N-acetylaspartate ratios in the right frontal white matter, indicative of decreased healthy neuronal population suffering significant oxidative stress. The basal ganglia did not show significant injury. He had left-sided hemiplegia with deficits in grasp, weightbearing, sitting, and transitional movements.

### 3.1 Feasibility and safety

All 3 infants were able to complete the full 40 h of intervention. The mean CIMT treatment duration was 114 ± 8 min per session. taVNS was triggered for 58 ± 15% of total CIMT time at a mean intensity of 0.57 mA. The youngest participant (Infant 1) required a rest break during most sessions. Infants 2 and 3 did not need extended breaks.

The infants tolerated the ear electrode, device set-up and stimulation well with no significant adverse events. Mild redness at the ear was noted 7 times out of a total of 120 sessions, but all redness resolved by the next day. Perceptual thresholds (PT) were determined by vocalizations or the child bringing their hand to their ear. When stimulation levels were decreased by 0.1 mA below the PT, the infants returned to typical activity levels and demeanor. Perceptual thresholds did occasionally change during 2 h sessions with the infants demonstrating a mid-session response to stimulation a total of 10 times during 120 sessions, but therapists were able to continue treatment at lower levels in all instances.

No other adverse responses were noted. All 3 parents reported an initial increase in infant fatigue after the first week with increased standard naps length or an earlier bedtime. Two of the three parents reported an increase in babbling and/or word usage in their daily reports.

The set-up added minimal time to therapy time. With the trigger closely wrapped to the hand, therapists were able to use both hands for activities, tactile cuing, and physical support. The wire connecting the trigger to the device broke twice in 120 h of treatment, which included regular use of swings, therapy balls, and dynamic gross motor activities.

### 3.2 CIMT fidelity and feasibility

**Figure 2:**
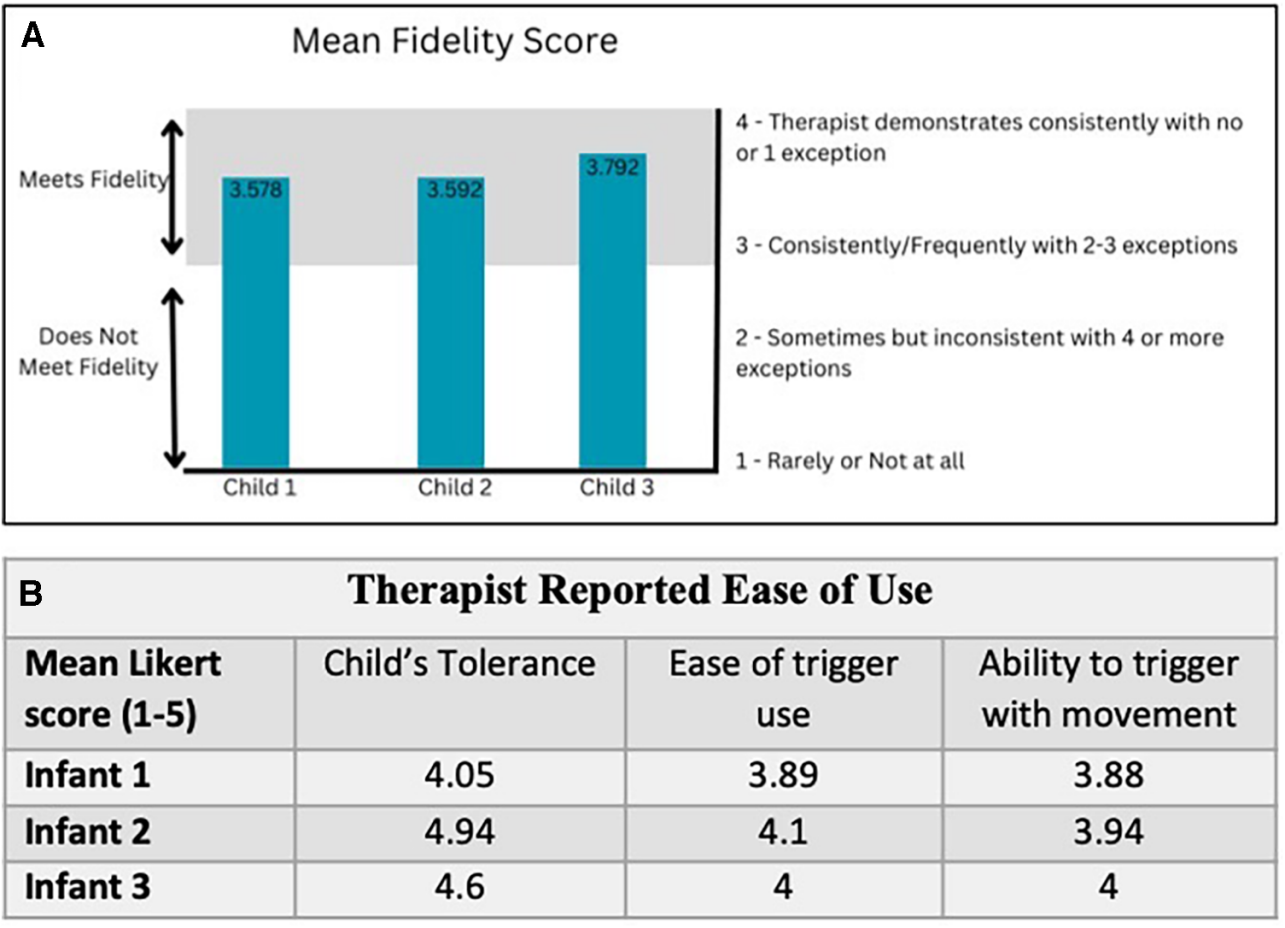
Fidelity and ease of use results.

The FIRM scores for 24 videos out of a total of 60 sessions ranged from 3.58–3.79. A fidelity score of 3 indicates adhering consistently to CIMT principles and a score of 4 is regarded as very high quality CIMT. There was an initial learning curve to incorporating the handheld trigger while attempting to maintain fidelity of CIMT. Subjectively, therapists reported improved accuracy with succeeding sessions. Therefore, taVNS was able to be added without compromising the quality of CIMT (Figure 2A).

At the end of each session the therapists rated their perception of (1) the infant's tolerance, (2) ease of trigger use and (3) their ability to accurately trigger the device with active movement. Averages of these ratings for each child are shown in (Figure 2B). The most frequent problem was wire management. Mental fatigue was associated with engaging a new participant and device management, but improved as sessions continued. Therapists agreed that on average, the child tolerated the stimulation and the device did not interfere with delivering CIMT.

### 3.4 Motor outcomes

**Figure 3:**
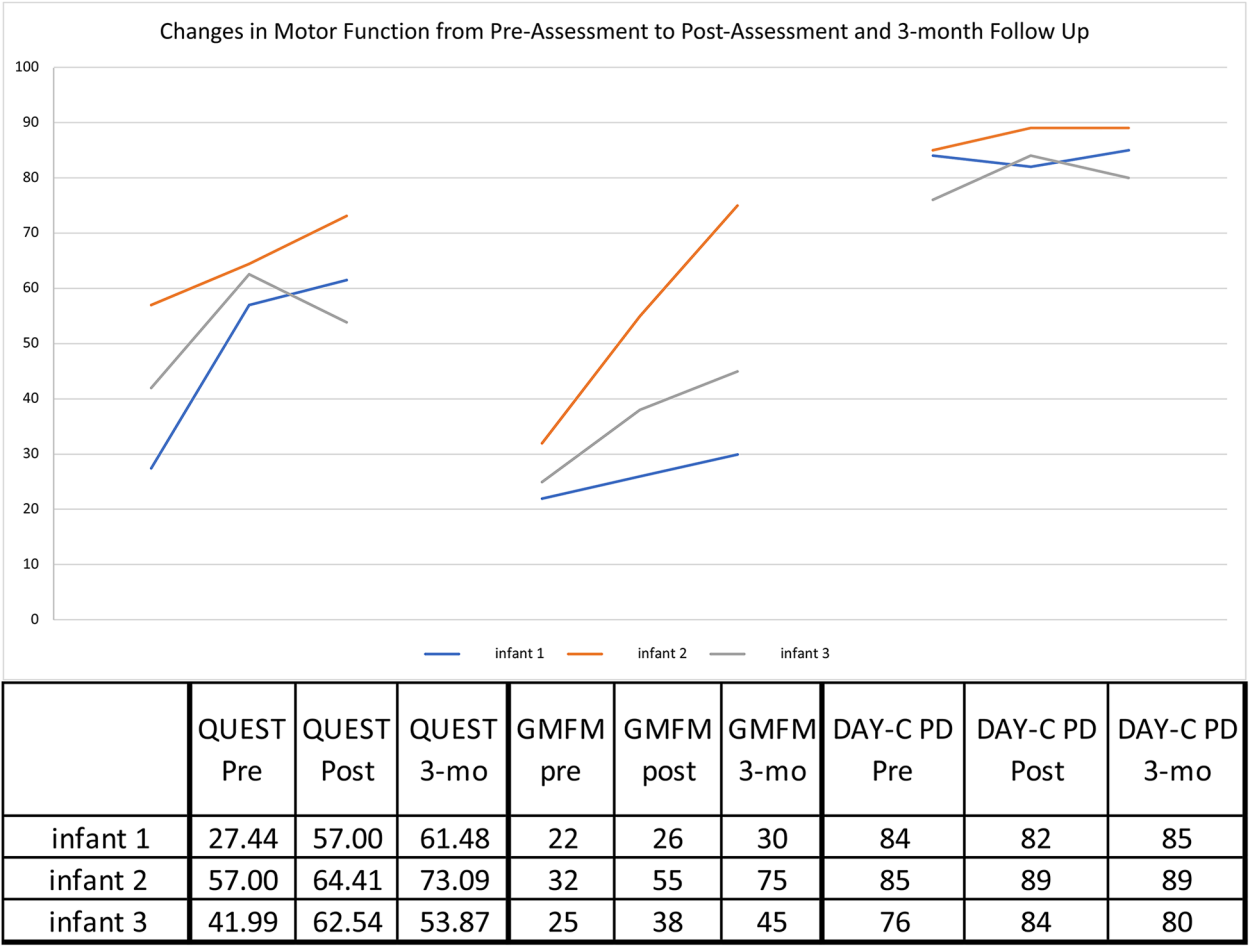
Motor outcome measures. GMFM—Gross Motor Function Measure—88 Interpreted in % value.; DAYC-2—Developmental Assessment of Young Children—2nd edition—standard scores shown; QUEST—Quality of Upper Extremity Skills Test—Summary Scores.

All three infants showed gains in upper extremity function as measured by the QUEST (Figure 3). From pre- to immediately post-treatment, all infants showed significant gains in the summary scores: Infant 1: +29.56, Infant 2: +7.41, and Infant 3: +20.55 points. All scores are well above the MCID of 4.89 points (34). The QUEST reports on two individual sections: dissociated movement and grasping. For dissociated movement, infant 1 increased +40.63, infant 2 had no change, and infant 3 increased +7.82 points. For grasping, infant 1 increased +18.5, infant 2 + 14.82, and infant 3 + 33.33 points. Infants 1 & 2 continued to make progress at follow-up at 3 months, but infant 3 decreased from post to 3 months follow-up, though still 11.88 points above baseline for the overall QUEST score.

Each infant showed gross motor gains from pre- to immediately post-treatment on the GMFM-88 (Figure 3), particularly Infant 2 (+23%) and Infant 3 (+13%) compared with MCID of 0.1%–3.0% (35) on this assessment. All infants continued to make progress from immediately post-treatment to the 3 month follow-up, and infants 2 & 3 had further gains of +20% and +7%, respectively.

For overall development, infants 2 and 3's DAYC-2 standard scores improved by 3 and 8 points, respectively, when compared to same aged peers. Infant 1 decreased from pre- to immediately post treatment by 1 point but then increased at 4 months post-treatment. Infant 2's score was maintained, but as with more specific motor tests, infant 3's score decreased by 4 points at the 3-month follow-up.

### 3.5 Goal attainment scaling

**Figure 4:**
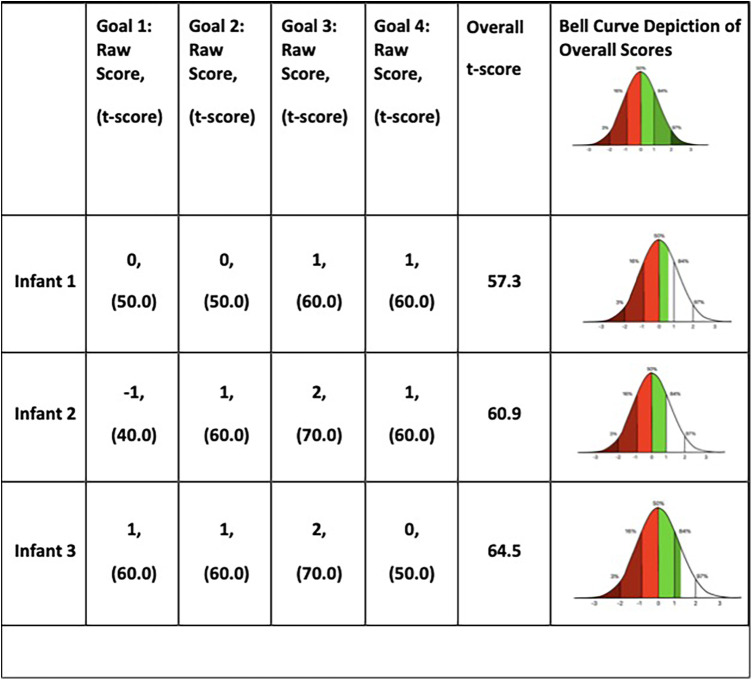
GAS outcomes.

All four goals were met or exceeded in each infant except for one goal with Infant 2. Infant 2 did not achieve the expected goal of “reaching to grasp a 1-inch block 9 times”, though he made progress from baseline. The three infants' overall t-scores ranged from 57.3– 64.5 indicating that they exceeded their expected outcomes at, or close to +1 (Figure 4). See Supplementary Table for more information on GAS goals.

## 4 Discussion

In this open-label pilot study of taVNS in 10–14 month infants with hemiplegia, we found that taVNS paired with CIMT is safe, feasible, and does not decrease the quality of treatment. Our preliminary data indicate that the improvement in infants' motor scores were greater than both the minimal clinically important difference and the improvement expected with a low dose of CIMT. All three infants exceeded the expected goals for therapy as measured by the goal attainment scores set with experienced therapists and parents. Although mentally taxing for the therapist initially, the fidelity of CIMT delivery while triggering taVNS was excellent for all infants and improved with the progression of treatment sessions. These results provide preliminary support for a proof of concept that combining taVNS + CIMT in infants may help improve upper extremity function in a short time when compared to CIMT alone. Further studies will be needed to establish the effectiveness of this novel combined intervention and the duration of optimal treatment for retention of motor skills.

While CIMT is the current gold standard for motor rehabilitation, based on decades of research, clinical penetration has been relatively low in infants. CIMT requires a high dosage to achieve functional motor changes which involves a time, transportation and resource commitment that is unfeasible for many families. CIMT is rarely covered by insurance, and families may pay thousands of dollars for CIMT their child even if they can find a program. Due to limited reimbursement, most clinics do not offer CIMT, and families may travel several hours daily to engage in a program. Therefore, caregivers must take significant time off from work and have reliable transportation, which limits CIMT to advantaged families. This creates a significant gap in equality, justice and benefit of this potentially effective therapy which must be addressed. Any additive treatment that decreases the time and resources required of families would ultimately extend CIMT therapy to less resource-rich families and promote more equal access in the home environment.

There is strong evidence in animal and adult rehabilitation literature that vagus nerve stimulation paired with active rehabilitation treatment promotes beneficial neuroplasticity leading to improved motor outcomes (36, 37). Our data and others suggest that taVNS may have similar benefits and is safe in vulnerable infant populations (12, 21, 31, 38–38). If future studies demonstrate motor improvements at lower doses of 40 h when taVNS is combined with CIMT in young infants, then these burdens will be lessened considerably, and the therapy can be made more widely available to families regardless of resources.

Prior research in older children with CP ages 3–10 provides a basis for expected changes with participation in CIMT programs (39). QUEST mean grasp scores changed by +11 points after 90 h of CIMT (6 h/day for 15 days). In our study in younger infants, the mean grasp score increased 22 points, even though the total treatment time was less than half that in previous studies of CIMT alone. Raw QUEST scores for each infant in our study continued to increase at the 3month assessment, but the scaled scores decreased in 2 infants relative to typically functioning peers. Possible reasons for this decrease in developmental trajectory are lack of adherence with the post-treatment transition plan at home and the fact that significant injuries such as with frontal or centrum semiovale white matter infarctions will require new connections be established to work around injured areas, which may result in difficulties in making the complex connections necessary to build on motor learning. This may be an ongoing process that necessitates longer duration of therapy or booster doses at intervals for some infants.

One large study (*n* = 145) investigated the effects of an 80-hour CIMT therapy program with children 3–7 years old (40). The mean gains of 3.46 to 8.86 in GMFM scores were negatively associated with the child's baseline GMFCS severity level. Our results with taVNS paired with CIMT also showed greater gains among those with less severe GMFM scores at baseline, but with similar or better change scores in younger infants who received half the total therapy time as the reference cohort. Infant 1, with severe hemiplegia, showed similar results to children with higher GMFCS IV level deficits (Δ = 4.0 compared to 3.46) (40). Infant 3, with moderately severe gross motor deficits, had Δ = 7.0 at post-treatment and further Δ of 13 at 3-months for a total Δ20, compared to Δ of 8.86 in children with GMFCS II level deficits (40). Infant 2, with moderate gross motor deficits, had >2 fold improvement in GMFCS: Δ23 immediately after treatment then further Δ20 at 3-months(total Δ 43), compared to Δ 8.86 in children with GMFCS II level deficits (40).

Infants age 6–12 months who were later diagnosed with CP showed a median *decrease* in DAYC scores of 23 points in 6 months (41). The DAYC has been used as an assessment to help in early identification of infants with cerebral palsy (41), but is not as sensitive for detecting a response to treatment. Nevertheless, the relative stability of scores for our infants over 3 months is a positive indicator that the taVNS paired with CIMT may help infants continue to make developmental gains relative to the infant's baseline function prior to starting this intensive therapy.

Adherence to CIMT treatment fidelity was excellent. Video review of sessions showed the therapists were able to time verbal and physical prompts accurately, select appropriate activities, reinforce correct behaviors, provide adequate repetition, and adapt tasks as needed. Importantly, the custom set-up did not limit the types of activities the therapist was able to do. They were still able to regularly work on balance using swings/therapy balls, climb on therapy equipment, and play in motorized toy cars. Refinements to the device setup and system of delivery may help lessen the therapist burden and allow delivery of taVNS paired with CIMT within the home setting.

### 4.1 Limitations

Limitations of this report include the open-label nature of this first-in-infants feasibility trial, small sample size, non-blinded raters, and lack of a control group. While informal parent/caregiver adherence to suggested home activities was documented during treatment, future studies should include a formal adherence follow-up plan with documentation to assess an individual family's abilities to follow through with home activities in the transition packages as this may significantly impact results at the 3-month follow-up.

## 5 Conclusion

Early data is promising that taVNS paired with intensive motor CIMT is feasible, reliable and safe in young infants with hemiplegia, and may help harness activity-dependent plasticity to enhance functional movement. Further studies will be needed to determine the optimal dosing and potential efficacy of taVNS paired with CIMT.

## Data Availability

The raw data supporting the conclusions of this article will be made available by the authors, without undue reservation.
