# Supplementary material for: Pairing taVNS and CIMT is feasible and may improve upper extremity function in infants
Source: Front Pediatr. 2024 Feb 13;12:1365767. doi: 10.3389/fped.2024.1365767 (PMC10896996; doi:10.3389/fped.2024.1365767)
Supplement: Supplementary file 1 [file Table1.pdf]

TVC01	
**Goal Description**	**Goal Attained**
Child will use 2 hands to bring a handled cup to mouth 0/5, 1/5, *2/5, 3/5, 4/5, 5/5 trials.	0
Child will transition sit to prone with total assistance, maximum assistance, *moderate assistance, minimum assistance, stand by assistance.	0
Child will sustain purposeful gross grasp for 0, 3, 6, *9, 12 seconds.	1
Child will initiate reach with his LUE, 1, 3, 5, *7, 9 times, in 2 minutes.	1
TVC02	
**Goal Description**	**Goal Attained**
Child will successfully supinate forearm past neutral to get food items to mouth during self-feeding with RUE 0, 2,4,*6, 8 trials in 10-minute feeding session	1
When in quadruped, child will crawl forward 4 paces with maximum assistance, moderate assistance, minimum assistance, tactile cues, *independently at RLE.	2
When placed into half kneeling position, child can pull to stand at bench with cues to position BUEs on top of bench with maximum assist, moderate assist, *minimum assist, contact guard assist, independently.	0
Child will combine a reach and grasp for an unsupported 1in toy with thumb and fingers opposed 3, *6, 9, 12, 15 times during play in a session	-1 (goal not met)
TVC03	
**Goal Description**	**Goal Attained**
Child will grasp a stabilized cylindrical item with thumb in opposition on 1/10, 3/10, 5/10, *7/10, 9/10 trials	1
Child will initiate active wrist extension past neutral with a forward reach at vertical surface 0%, 25%, 50%, *75%, 100% of trials	1
In independent ring sit, child will reach to retrieve toy at feet and re-erect 0, 1, 2, 3, *4 times independently.	2
Child will lower from sitting to prone with trunk control and demonstrate weightbearing on extended wrist with maximum assist, moderate assist, *minimal assist, tactile cues, independently.	0

***outcome achieved**
